# Advances in Vibrational Spectroscopic Techniques for the Detection of Bio-Active Compounds in Virgin Olive Oils: A Comprehensive Review

**DOI:** 10.3390/foods13233894

**Published:** 2024-12-03

**Authors:** Fangchen Ding, Sebastián Sánchez-Villasclaras, Leiqing Pan, Weijie Lan, Juan Francisco García-Martín

**Affiliations:** 1 Departamento de Ingeniería Química, Facultad de Química, Universidad de Sevilla, 41012 Sevilla, Spain; fandin@alum.us.es; 2 University Institute of Research on Olive Grove and Olive Oils, GEOLIT Science and Technology Park, University of Jaen, 23620 Mengibar, Spain; 3 College of Food Science and Technology, Nanjing Agricultural University, No. 1, Weigang Road, Nanjing 210095, China; pan_leiqing@njau.edu.cn (L.P.); weijie.lan@njau.edu.cn (W.L.)

**Keywords:** bio-active compounds, chemometric techniques, rapid analysis, vibrational spectroscopy, virgin olive oils

## Abstract

Vibrational spectroscopic techniques have gained significant attention in recent years for their potential in the rapid and efficient analysis of virgin olive oils, offering a distinct advantage over traditional methods. These techniques are particularly valuable for detecting and quantifying bio-active compounds that contribute to the nutritional and health benefits of virgin olive oils. This comprehensive review explores the latest advancements in vibrational spectroscopic techniques applied to virgin olive oils, focusing on the detection and measurement of key bio-active compounds such as unsaturated fatty acids, phenolic compounds, and other antioxidant compounds. The review highlights the improvements in vibrational spectroscopy, data processing, and chemometric techniques that have significantly enhanced the ability to accurately identify these compounds compared to conventional analytical methods. Additionally, it addresses current challenges, including the need for standardized methodologies and the potential for integrating vibrational spectroscopy with other analytical techniques to improve accuracy and reliability. Finally, findings over the last two decades, in which vibrational spectroscopy techniques were effectively used for the detailed characterization of bio-active compounds in virgin olive oils, are discussed.

## 1. Introduction

Virgin olive oil (VOO), as a typical high-quality natural product of the Mediterranean region, refers to oils obtained from the fruit of the olive tree (*Olea europaea* L.) solely by mechanical or other physical means under conditions, particularly thermal conditions, that do not lead to alterations in the oil, and which have not undergone any treatment other than washing, decantation, centrifugation, and filtration [1]. Studies have demonstrated that the high consumption of VOO, as the main source of fat in the Mediterranean diet, is an effective factor in reducing the risk of cardiovascular diseases, strokes, and certain types of cancer [2,3,4]. This is primarily attributed to the bio-active compounds present in olive oil, including phenolic substances, fatty acids (mainly oleic acid), and various antioxidants such as tocopherols, squalene, chlorophyll, carotenoids [5,6,7], and others.

Regarding bio-active compounds, a precise definition has yet to be established. However, there is a consensus among researchers that these compounds have the ability to interact with certain components of living tissues and elicit a wide range of biological effects [8,9,10]. Therefore, the bio-active compounds in VOO are considered a significant source of its health benefits.

In recent years, VOO has gained recognition as a functional food, leading to an expanding global consumption market [11] and a gradual increase in annual demand. This trend can be attributed to the increasing awareness among today’s consumers of the health benefits of olive oil [12] and the continuous exploration of the mechanisms by which the bio-active components in olive oil impact human health. For example, the phenolic compounds in VOO, particularly oleuropein and hydroxytyrosol, exhibit high antioxidant activity, which can protect biological lipids, proteins, and DNA molecules by significantly scavenging free radicals, thereby delaying the progression of neurodegenerative diseases [13,14]. Additionally, numerous studies have demonstrated that the phenolic substances in olive oil also have preventative effects against atherosclerosis, lower blood cholesterol levels, and possess antibacterial and anti-inflammatory properties [15,16,17,18]. In addition to phenolic compounds, the unsaturated fatty acids (UFA) in VOO, particularly oleic acid and linoleic acid, have been found by researchers to influence cancer onset and metastasis by inhibiting the overexpression of the oncogene HER2 [19]. These UFAs also exhibit antithrombotic and blood pressure-lowering effects [20,21]. However, it must be mentioned that VOO also contains trace antioxidants in forms other than phenolic compounds that exhibit similar antioxidant activities, such as tocopherols, squalene, and pigments (e.g., chlorophyll and carotenoids), and these have also been demonstrated to possess significant capabilities in inhibiting oxidative stress in organisms, thereby contributing to the prevention of certain types of cancer to some extent [22]. Particularly, squalene in olive oil has been demonstrated to possess antitumor effects against colon and lung cancer [23,24]. Regarding the pigments in olive oil, they are commonly recognized for their influence on the color of oil and are used as indicators of its quality. However, it is noteworthy that the intake of these pigments is also significantly correlated with the incidence of certain types of cancer. For instance, the associations between six common carotenoids and the ten most prevalent types of cancer have been reviewed, ultimately suggesting that increasing the intake of carotenoids may be an effective method for reducing cancer risk [25]. Similarly, lycopene, as a type of carotenoid, is frequently used in the prevention of breast and prostate cancers and in inhibiting cancer progression [26,27].

Given the significant health benefits of bio-active components in olive oil, increasing research efforts have focused on accurately characterizing these components. This is undoubtedly a challenging task due to the very low concentrations of certain bio-active compounds in olive oil. For instance, the total phenolic content in virgin olive oil (VOO) is approximately 100 mg/kg [28]. Specifically, hydroxytyrosol and tyrosol, the two most abundant phenolic compounds in VOO, have concentrations ranging from 50 to 200 mg/kg and 40 to 180 mg/kg [29], respectively. In contrast, oleuropein, another biologically active phenolic compound, is found only in the range of 0.1–0.5 mg/kg [30]. Numerous studies have been published on the traditional methods for determining the bio-active components in VOO. Recently, complex selective techniques such as high-performance liquid chromatography (HPLC) coupled with fluorescence detector (FLD) [31,32], photodiode array (PDA) [33,34], nuclear magnetic resonance (NMR) [35,36] or mass spectrometry (MS) [37,38,39] and, in the latest advancements, time-of-flight mass spectrometry (TOFMS) [40,41,42] have been developed for the identification and quantification of phenolic compounds, unsaturated fatty acids, tocopherols, squalene, and other types of antioxidant bio-active components in VOO. The traditional detection techniques mentioned above, while effective, are often time-consuming and costly, rendering them unsuitable for high-throughput, large-scale rapid detection. However, with ongoing advancements in optical technology, materials science, and computer science, the use of vibrational spectroscopy combined with chemometrics or nanomaterial-enhanced methods has become increasingly sophisticated. These advancements allow for the quantitative characterization of low-concentration bio-active substances with greater efficiency and precision.

In this paper, three vibrational spectroscopy techniques, namely near-infrared (NIR) spectroscopy, mid-infrared (MIR) spectroscopy, and Raman spectroscopy are comprehensively reviewed in their use for the identification and quantitative detection of bio-active components in VOO, including comparisons of the three techniques along with the advantages and disadvantages of each one. Although far-infrared spectroscopy, also known as terahertz spectroscopy [43], is widely used in food analysis, its application in detecting active compounds in olive oil remains notably limited. Regarding hyperspectral imaging, due to the high transmittance of oil products, significant scattering and absorption of light occur as it propagates through the liquid. This phenomenon adversely affects the quality of hyperspectral imaging. Therefore, despite being forms of vibrational spectroscopy, far-infrared spectroscopy and hyperspectral imaging will not be discussed in this paper. The aims of this review are as follows:

(1) To outline the characteristics, applications, potential, and limitations of these three vibrational spectroscopy techniques for characterizing bio-active components in VOO.
(2) To present examples of how these techniques are applied in both industrial and laboratory settings for the analysis of bio-active components in VOO.
(3) To discuss the principles related to chemometric techniques and nanomaterial signal enhancement, improving the understanding of spectral data analysis and the application of various signal enhancement techniques in the detection of low-concentration bio-active components.

In the introduction, the primary bio-active components found in VOO and their beneficial effects on human health are outlined. Additionally, an overview of traditional methods used to detect bio-active components in VOO is provided and their limitations are discussed. In the subsequent sections, the applications and principles of various vibrational spectroscopy methods for detecting bio-active components in VOO and other seed oils are explored. Finally, the strengths and weaknesses of these techniques in practical applications are analyzed and their potential to improve detection accuracy and efficiency is assessed.

## 2. Vibrational Spectroscopic Techniques

In recent years, vibrational spectroscopy has garnered significant attention and development in the quality assessment of oil products, particularly olive oil. Techniques like NIR, MIR, and Raman spectroscopy have proven effective in rapidly and accurately evaluating critical quality indicators such as acidity, peroxide value, and fatty acid composition, which directly impact the sensory and nutritional qualities of the oil. Raman spectroscopy, with its minimal sample preparation and ability to distinguish similar compounds, is widely used to detect adulteration in olive oil, ensuring consumer protection.

Beyond basic quality assessment, vibrational spectroscopy also shows great potential in the identification and quantification of bio-active compounds in olive oil. For instance, the high antioxidant content in olive oil, including polyphenols like hydroxytyrosol and oleuropein, contributes to both the stability of the oil and its significant health benefits. Vibrational spectroscopy enables the precise analysis of the concentrations and structural variations of these compounds, thereby providing a robust scientific foundation for the certification of quality and the functional evaluation of olive oil.

Furthermore, the efficiency, non-destructive nature, and cost-effectiveness of vibrational spectroscopy make it an ideal alternative to traditional wet chemistry methods [44]. These techniques are particularly valuable in real-time, online monitoring, offering accurate analytical data that greatly enhance quality control during production processes [45]. This applicability extends not only to the quality assurance of olive oil but also to a broad range of other oil products and agricultural commodities.

In summary, vibrational spectroscopy demonstrates a strong potential in oil quality assessment, especially in the characterization of olive oil and bio-active compound detection. As these technologies continue to advance, they are expected to further improve the precision and efficiency of oil quality testing, providing more reliable tools for quality control and safety management in the food industry.

## 3. Near-Infrared Spectroscopy (NIRS)

NIRS is a spectroscopic technique based on the principle of molecular vibration. The basic principle is that when NIR light (usually in the wavelength range of 780 to 2500 nm) is irradiated onto a sample, the molecules within absorb light energy at specific wavelengths. This absorption causes transitions in the vibrational energy levels of the molecules, corresponding to the vibrational modes of chemical bonds such as C–H, N–H, and O–H. By analyzing the intensities of these absorption peaks, valuable information about the molecular vibrational composition can be deduced [46]. Moreover, NIR spectroscopy offers rapid spectral acquisition with minimal or no sample preparation, allowing for the analysis of samples across various matrix types, including solids, powders, films, gels, and liquids. The development of miniaturized NIR devices further enhances the versatility of technique, enabling on-site measurements without the need to collect samples for subsequent laboratory analysis [47].

**Figure 1:**
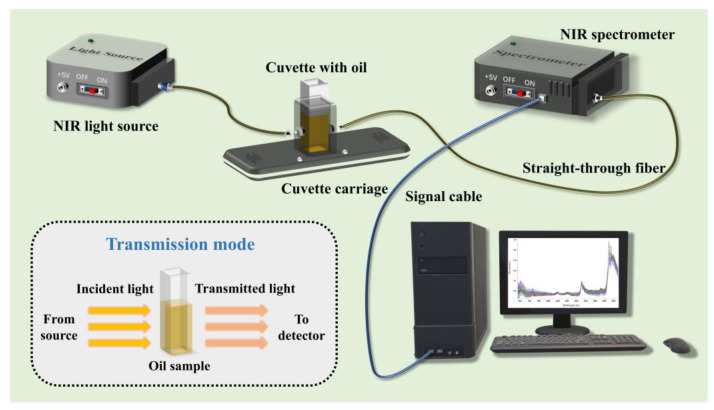
Common transmission modes used in NIRS detection of oil products.

Regarding the acquisition of NIR spectra, the main modes of acquisition include transmittance, reflectance, and transflectance. For solid or non-homogeneous samples, reflectance is generally acquired using a Y-fiber probe. For homogeneous oils, especially olive oil, transmittance measurements based on Beer Lambert’s law [48] are typically performed with a straight-through optical fiber and cuvettes of different path lengths (Figure 1). It has been shown that increasing the path length during spectral acquisition enhances the prominence of NIR absorption peaks in olive oils, while the positions of these peaks remain unchanged [49]. Additionally, significant differences in the performance of quantitative regression models for compounds based on NIR spectra collected at different path lengths were also not observed. In this sense, conducting a thorough analysis of the standard NIR spectra of olive oil is crucial. This foundational understanding enables the accurate identification of key spectral features, which is essential for developing reliable predictive models for various compounds in olive oil.

**Figure 2:**
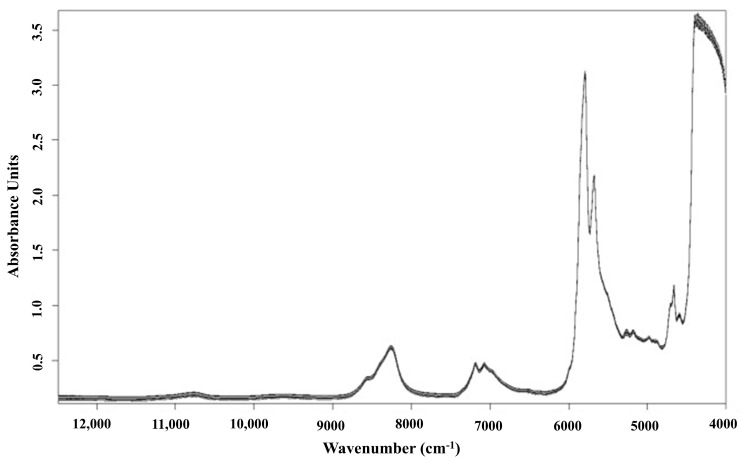
NIR spectra of VOO (Figure adapted from [57]).

Figure 2 illustrates the typical NIR spectra of VOO in the NIR region of 800–2500 nm (12,500–4000 cm^−1^), and Table 1 shows the main selected variables for NIR which were associated with the absorption bands of the most important compounds of olive oil, like fatty acids, phenols, squalene, and tocopherols. Absorption bands were observed at 1208 nm (8278 cm^−1^), which corresponds to the second overtone of the C–H stretch [50]. Additionally, bands at 1720 and 1760 nm (5814 and 5681 cm^−1^) were linked to the first overtone of the C–H stretch in various chemical groups, including methyl, methylene, and vinyl [51,52]. The band at 1720 nm (5814 cm^−1^) is the same as that of the triolein spectrum, which has been reported to be observed at 1725 nm (5797 cm^−1^) [53]. An absorption band at 2144 nm (4664 cm^−1^) is associated with the combination tone of C=C and C–H stretch in *cis* unsaturated fatty acids, while the C–H stretch bands around 2100 nm (4762 cm^−1^), characteristic of terminal double bonds and *cis* unsaturation [54]. The figure also shows broad bands at around 1400 and 1950 nm (7143 and 5128 cm^−1^) related to the O–H first overtone and to a combination band, respectively, of water [47,55], and the C–H stretch first overtone of carbonyl compounds at 1832 nm (5458 cm^−1^) [54,56]. 

After comprehending the functional groups in VOO that contribute to NIRS absorption peaks, it is equally important to recognize the limitations of NIRS in practical applications. This awareness aids in the more accurate interpretation of spectral data and helps to avoid potential errors when developing chemometrics models. Furthermore, acknowledging the limitations of NIRS encourages the integration of complementary analytical methods to validate and enhance NIRS results, thereby improving the overall precision and reliability of analyzing the intrinsic components of olive oil. Firstly, the absorption bands in NIR spectra are typically broad and overlapping, leading to lower spectral resolution, which complicates the differentiation among various compounds [60]. Secondly, NIRS is highly sensitive to water absorption, which can interfere with the detection of specific compounds [61]. For example, absorption bands around 1400 nm and 1950 nm (7143 and 5128 cm^−1^) arise from the first overtone of the stretching vibration of the abundance of O–H groups in hydroxytyrosol and tyrosol while those bands are also associated with water. This was the primary reason why NIRS could not be used to accurately predict the levels of hydroxytyrosol and tyrosol in VOO [62]. Additionally, the weak absorption signals of certain trace components in NIR spectra present challenges in their detection [63], often requiring the use of advanced mathematical processing and correction techniques to improve accuracy. However, with the rapid advancement of chemometrics, many of the limitations of NIRS are gradually being addressed. The integration of chemometrics with advanced algorithms and statistical modeling has enabled more accurate and efficient analysis of complex spectral data. Techniques such as multivariate data analysis, partial least squares (PLS), and principal component analysis (PCA) have proven effective in separating overlapping absorption peaks [64,65,66], thereby enhancing the differentiation and detection of various compounds. These methods not only improve the accuracy of NIR spectroscopy in the analysis of multi-component samples but also extend its applicability to the detection of trace components.

Table 2 presents recent studies that have employed NIRS combined with chemometrics for the quantification of bio-active components in VOO, highlighting the variation in predictive model performance across different substances. For instance, NIRS was employed to quantify tocopherols in VOO [55]. However, the prediction of β--tocopherol, which is a minor component, was poor (R_cv_ less than 0.6) due to its low concentrations in VOO (0.7–103.8 mg/kg). In contrast, the predictive model for α-tocopherol, the main tocopherol in VOO with concentrations ranging from 54.5 to 755.9 mg/kg, performed satisfactorily (R_cv_ = 0.91). Meanwhile, the prediction models for chlorophylls and carotenoids content in VOO have similarly shown poor performance [62,67,68]. This outcome is primarily due to the fact that these pigments, which are responsible for the characteristic green and yellow colors of olive oil, exhibit strong absorption in the visible light bands between 430–480 nm and 640–660 nm, rather than solely because of their low concentrations in VOO. Conversely, most NIR models for unsaturated fatty acids (oleic, linoleic, and linolenic acid) in VOO have demonstrated robust performance, with R_v_^2^ values exceeding 0.85, which indicated strong model reliability [62,69,70,71]. This success is not only attributed to the high concentrations of unsaturated fatty acids in VOO but also to the presence of numerous C–H stretching vibrations within their molecular structures, which result in strong spectral absorption [58], making these fatty acids more readily and accurately predictable. It should be noted that the NIR spectra acquisition in all of the studies cited in Table 2 were carried out in transmittance mode.

Although many of the studies mentioned above have successfully employed NIRS for the quantitative prediction of bio-active components in VOO, it is evident that the full potential of NIRS has yet to be fully realized. To begin with, the sample size, geographical origin, and varietal diversity of VOO samples should be as extensive as possible to ensure that the models developed are robust and generalizable across different conditions. Furthermore, in the preprocessing of NIRS data, few researchers have utilized techniques such as spectral variable selection to optimize model performance, reduce noise interferences, and enhance the detection sensitivity for target compounds. The implementation of such methods could significantly improve the accuracy and reliability of the predictions [74]. Moreover, nearly all studies have relied on PLS models, which are traditional and powerful algorithms with broad applicability, and are insufficient on their own to support the complex demands of quantitative and classification analysis for specific food types or components in the interdisciplinary field of chemometrics. As deep learning techniques (such as neural networks) continue to evolve and mature, they present a promising avenue for the detection of specific components in food, particularly for the bio-active substances in olive oils. The application of deep learning models, with their ability to capture complex patterns and interactions within large datasets, should be explored to push the boundaries of what NIRS can achieve in the accurate and sensitive detection of these valuable compounds.

**Figure 3:**
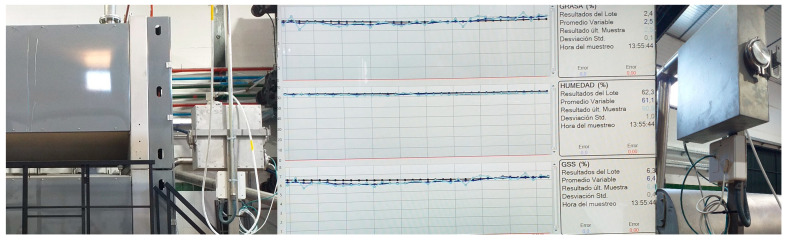
Online monitoring by NIRS of moisture and fat content on dry and wet basis in pomace from two-outlet decanters.

Finally, it is worth noting that there is a wide range of commercial NIRS equipment available both for research laboratories and for laboratories that support olive mills and farmers. This allows them to characterize the olive fruit, olive paste, olive pomace, and more recently (although to a lesser extent), the quality parameters of olive oils [47]. In addition, at the olive mill level, NIRS is also used for the online monitoring of the depletion of the pomace by measuring its fat and moisture content (Figure 3). This is truly a major innovation at the mill level.

## 4. Mid-Infrared Spectroscopy (MIRS)

MIR spectroscopy refers to the infrared spectral region with wavenumbers ranging from 4000 to 400 cm^−1^, corresponding to wavelengths between 2.5 and 25 μm. Within this range, the spectrum is further divided into two distinct regions: the functional group region and the fingerprint region [75]. Absorption peaks in these two regions correspond to different molecular vibrational modes, aiding researchers in identifying and characterizing functional groups and the overall structure of molecules [76]. The functional group region typically falls within the wavenumber range of 4000–1500 cm^−1^ (2.5–6.7 μm). In this region, absorption peaks associated with characteristic functional groups, such as O–H, N–H, C–H, and C=O, are generally strong, broad, and located at well-defined positions, making them suitable for qualitative analysis [77]. The fingerprint region, on the other hand, usually lies within the wavenumber range of 1500–400 cm^−1^ (6.7–25 μm). In this region, molecular vibrational modes are more complex, often involving skeletal vibrations such as the bending and stretching of C–C, C–O, and C–N bonds. These vibrational modes are closely related to the overall molecular structure, and the absorption peaks in this region are often more specific, making them ideal for molecular identification and structural analysis.

Compared to NIRS, which primarily relies on overtones and combination frequency absorption and thus contains relatively less information, MIRS offers more detailed functional group and structural information because it directly probes the fundamental vibrational modes of molecules [78]. The high specificity of the functional group region combined with the complexity of the fingerprint region makes MIRS a powerful tool for molecular identification and qualitative analysis. However, MIRS also has disadvantages, including the need for complex sample preparation and longer spectral acquisition times. To address these challenges, MIRS is often coupled with Fourier Transform (FT) technology for compound analysis. Traditional infrared spectrometers obtain spectra by sequentially scanning with monochromatic light, while Fourier Transform infrared (FT-IR) uses an interferometer to simultaneously expose the sample to all infrared frequencies, recording an interferogram in the time domain [79]. The Fourier Transform of the interferogram then produces the spectral information of the sample. Compared to the traditional monochromatic scanning method, FT-IR significantly speeds up spectral acquisition and allows for higher resolution by adjusting the optical path difference of the interferometer [80], thereby better distinguishing closely spaced absorption peaks.

In FT-IR analysis, the selection of accessories is crucial for the analysis of specific samples, as it directly affects the measurement methods and data analytical precision. The attenuated total reflection (ATR) accessory is one of the preferred tools for analyzing the quality of olive oil. The ATR accessory operates by making contact with the sample surface and utilizing total internal reflection within a high-refractive-index crystal to obtain spectral data [81]. The primary advantage of ATR lies in its simplicity; it requires no complex preparation of the olive oil sample. The sample can be directly placed on the crystal surface, and high-quality spectral data can be obtained in a short amount of time. This convenience makes ATR particularly suitable for the rapid screening and quality control of large batches of samples. Additionally, ATR is capable of detecting trace components on the sample surface, allowing it to sensitively capture minor changes in the content of bio-active substances during the storage and processing of oils [82].

**Figure 4:**
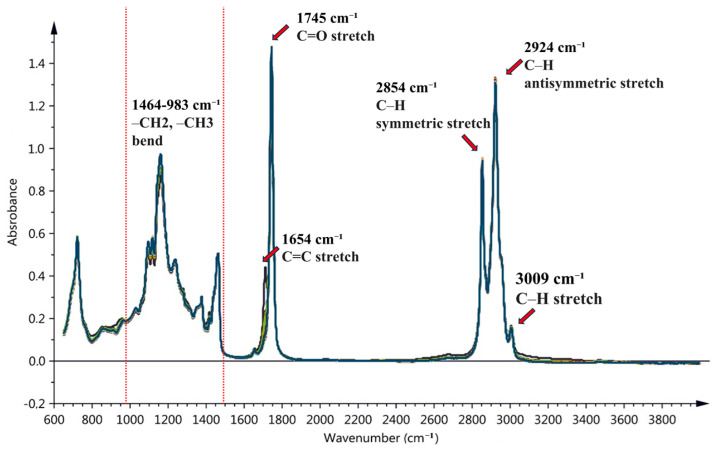
The positions of several key absorption bands in the MIRS region (Figure adapted from [83]).

Similar to NIRS, accurately interpreting spectra in MIRS analysis remains a major challenge. Table 3 and Figure 4 highlight the critical absorption bands in the MIR spectra of VOO. The complexity of MIR spectra makes both the quantitative and qualitative analysis of these absorption bands particularly challenging.

In MIRS analysis, data preprocessing and multivariate statistical analysis are critical steps to ensure the accurate interpretation of spectral information, especially when detecting bio-active compounds in virgin olive oils. MIRS spectral data often contain complex overlapped signals, baseline drifts, and noise. To mitigate these interferences and enhance the relevant characteristic absorption bands, preprocessing techniques such as Savitzky–Golay (SG) smoothing, standard normal variate (SNV), and orthogonal signal correction (OSC) are widely employed.

**Table 3:** MIRS absorption bands for a range of bonds important for bio-active compound analysis in VOO (table adapted from other research [58,63,84,85]).

Bond	Compound/Functional Group	Wavenumber (cm^−1^)
O–H stretch	Water, alcohol	3600–3200
C–H stretch	Alkenes	3100–3000
C–H stretch	Aromatic ring	3060–3020
C–H stretch	Methylene group	2960–2860
C=O stretch	Carboxylic acids	~1743
C=O stretch	Saturated aldehydes	1750–1715
C=O stretch (amide I)	Amides	1700–1600
C=C stretch	Alkenes	1666–1640
C=C stretch	Aromatic ring	1625–1590, 1590–1575, 1525–1470, 1409–1425
C–H asymmetric stretch	Methyl and methylene groups	~1460
O–H deformation	Phenolic compounds	1390–1330
C–O-H deformation	Phenolic compounds	1382–1317
C–O vibration	Alkyl-aryl ether	1310–1210, 1120–1020
C–O stretch	Phenolic compounds	1260–1180
C–C stretch	Phenyl carbon	1225–1075
C–O and O-H stretch	Aromatic and alcohol	1230–1030
–C–H rocking vibration	Methoxy group	1211–1147
C–O stretching vibration	Phenolic compounds	1150–1040
C–H out-of-plane deformation	Aromatic ring	900–700
O–H out-of-plane deformation	Aromatic ring	~720

In the analysis of preprocessed MIRS data, partial least squares regression (PLSR) and principal component regression (PCR) are two widely used multivariate statistical methods, both of which have demonstrated exceptional performance in interpreting complex spectral data and quantifying bio-active compounds in VOO. The primary advantage of PLSR lies in its ability to simultaneously handle the regression relationship between characteristic variables (MIRS data) and object variables (bio-active compounds concentrations) by extracting latent variables (principal components) [86]. On the other hand, PCR first reduces the dimensionality of the original spectral data through principal component analysis (PCA), extracting a few principal components that explain the most variance in the data [87]. These principal components are then used as new predictor variables in a regression analysis to predict the concentrations of bio-active compounds in VOO.

Table 4 highlights several studies utilizing MIRS to detect bio-active compounds in virgin olive oil (VOO). In some of these studies [84,88,89,90], the combination of PLSR and MIRS demonstrated a strong ability to predict total phenolic content, with R^2^ values over 0.87, despite the researchers employing different spectral preprocessing methods. However, the quantitative prediction of individual phenolic compounds has been less satisfactory in some cases. For example, PLSR models developed for compounds such as hydroxytyrosol, tyrosol, vanillic acid, and syringic acid [72,90] failed to achieve R^2^ values above 0.7, with most models showing R^2^ values below 0.5. The researchers did not specify the reasons for the poor performance of these models. In contrast, the models developed by [89] for *ortho*-diphenols and flavonoids, based on PLSR and PCR, performed exceptionally well, with R_v_^2^ values exceeding 0.98. This discrepancy suggests that MIRS technology may exhibit significant variability in its effectiveness for the quantitative detection of different individual phenolic compounds. Given that the concentration ranges of the target compounds across these studies were similar, and that the spectral preprocessing methods and regression algorithms used were also comparable, the marked difference in predictive performance can likely be attributed to the substantially larger sample size used by [89], who utilized 449 samples. This larger sample size likely reduced uncertainties arising from sample heterogeneity or intra-sample variability, thereby enabling a more accurate capture of the concentration variations of the target compounds and their corresponding spectral features. Conversely, the studies by [72,90], which employed relatively smaller sample number (64 and 93 samples, respectively), may have failed to adequately cover the concentration range of the target compounds. This limited coverage could have resulted in insufficient capture of the spectral features specific to these compounds, subsequently affecting the predictive accuracy of the models. These findings underscore the importance of considering sample size and data distribution quality as critical factors in the development and validation of chemometric models, ensuring the robustness and broad applicability of the models.

**Table 4:** Developed chemometrics models for various bio-active compounds of VOO using different unit ranges, MIR spectral ranges, and spectral pre-processing methods.

Analytes	Units	Range	Sample Size	Spectral Acquisition	Wavelength Range (cm^−1^)	Spectral Preprocessing	Statistical Methods	Results	Reference
TPC	mg/kg	46–877	127	ATR	3610–816	–	PLSR	R_c_^2^ = 0.87 RMSEC = 22.4	[84]
TPC	mg GAE/g	0.39–1.72	449	ATR	4000–500	SNV	PLSR	R_v_^2^ = 0.95 RMSECV = 5.04	[89]
TPC	mg GAE/g	0.39–1.72	449	ATR	4000–500	SNV	PCR	R_v_^2^ = 0.99 RMSECV = 6.99	[89]
TPC	mg/kg	188.46–491.95	64	ATR	4000–650	SD	PLSR	R_c_^2^ = 0.99 RMSEC = 6.06	[90]
TPC	mg/kg	3.3–13.3	104	ATR	4000–700	2DSG	PLSR	R_v_^2^ = 0.97 RMSEV = 0.59	[88]
TPC	mg/kg	13.4–946.7	93	ATR	4000–375	FD + SNV	PLSR	R_v_^2^ = 0.44 RMSEV = 162.10 RPD = 1.13	[72]
Hydroxytyrosol	mg/kg	0.3–42.9	93	ATR	4000–375	FD + SNV	PLSR	R_v_^2^ = 0.17 RMSEV = 9.96 RPD = 1.35	[72]
Hydroxytyrosol	mg/kg	0.09–30.72	64	ATR	4000–650	SD	PLSR	R_cv_^2^ = 0.68 RMSECV = 4.66	[90]
Tyrosol	mg/kg	1.2–32.8	93	ATR	4000–375	FD + SNV	PLSR	R_v_^2^ = 0.32 RMSEV = 4.98 RPD = 1.34	[72]
Tyrosol	mg/kg	0.73–44.19	64	ATR	4000–650	SD	PLSR	R_cv_^2^ = 0.52 RMSECV = 7.97	[90]
Hydroxytyrosol secoiridoids	mg/kg	40.54–75.20	93	ATR	4000–375	FD + SNV	PLSR	R_v_^2^ = 0.19 RMSEV = 106.1 RPD = 0.97	[72]
Tyrosol secoiridoids	mg/kg	61.10–456.10	93	ATR	4000–375	FD + SNV	PLSR	R_v_^2^ = 0.30 RMSEV = 105.7 RPD = 0.98	[72]
Caffeic acid	mg/kg	0.01–0.60	64	ATR	4000–650	SD	PLSR	R_cv_^2^ = 0.24 RMSECV = 0.09	[90]
p-coumaric acid	mg/kg	0.02–8.13	64	ATR	4000–650	SD	PLSR	R_cv_^2^ = 0.36 RMSECV = 1.06	[90]
Vanillic acid	mg/kg	0.01–1.14	64	ATR	4000–650	SD	PLSR	R_cv_^2^ = 0.31 RMSECV = 0.16	[90]
Syringic acid	mg/kg	0.01–0.38	64	ATR	4000–650	SD	PLSR	R_cv_^2^ = 0.19 RMSECV = 0.06	[90]
Cinnamic acid	mg/kg	0.01–0.41	64	ATR	4000–650	SD	PLSR	R_cv_^2^ = 0.19 RMSECV = 0.07	[90]
Vanillin	mg/kg	0.01–1.14	64	ATR	4000–650	SD	PLSR	R_cv_^2^ = 0.31 RMSECV = 0.16	[90]
Apigenin	mg/kg	0.04–5.29	64	ATR	4000–650	SD	PLSR	R_cv_^2^ = 0.39 RMSECV = 0.92	[90]
Luteolin	mg/kg	0.02–2.55	64	ATR	4000–650	SD	PLSR	R_cv_^2^ = 0.08 RMSECV = 0.52	[90]
*Ortho*-diphenols	mg GAE/g	0.37–0.83	449	ATR	4000–500	SNV	PLSR	R_v_^2^ = 0.99 RMSECV = 8.05	[89]
*Ortho*-diphenols	mg GAE/g	0.37–0.83	449	ATR	4000–500	SNV	PCR	R_v_^2^ = 0.99 RMSECV = 7.69	[89]
Flavonoids	mg GAE/g	0.78–1.96	449	ATR	4000–500	SNV	PLSR	R_v_^2^ = 0.99 RMSECV = 5.28	[89]
Flavonoids	mg GAE/g	0.78–1.96	449	ATR	4000–500	SNV	PCR	R_v_^2^ = 0.98 RMSECV = 3.81	[89]
Oleic acid	%	62.0–80.0	86	ATR	4000–700	FD + SD	PLSR	R_v_^2^ = 0.92	[91]
Oleic acid	%	0.46–1.07	47	ATR	4000–650	OSC + WA	PLSR	R_cv_^2^ = 0.93 RMSECV = 0.97	[92]
Oleic acid	mg/kg	65.66–76.59	64	ATR	4000–650	SD	PLSR	R_v_^2^ = 0.94 RMSECV = 0.97	[90]
Oleic acid	mg/kg	29.9–78.0	104	ATR	4000–700	2DSG	PLSR	R_v_^2^ = 0.99 RMSEV = 1.41	[88]
Linoleic acid	%	5.3–15.0	86	ATR	4000–700	FD + SD	PLSR	R_v_^2^ = 0.94	[91]
Linoleic acid	%	0.12–0.83	47	ATR	4000–650	OSC + WA	PLSR	R_cv_^2^ = 0.93 RMSECV = 0.66	[92]
Linoleic acid	mg/kg	4.90–15.13	64	ATR	4000–650	SD	PLSR	R_cv_^2^ = 0.91 RMSECV = 0.76	[90]
Linoleic acid	mg/kg	5.7–41.0	104	ATR	4000–700	2DSG	PLSR	R_v_^2^ = 0.98 RMSEV = 1.40	[88]
Linolenic acid	%	0.44–0.83	47	ATR	4000–650	OSC + WA	PLSR	R_cv_^2^ = 0.64 RMSECV = 0.07	[92]
Linolenic acid	mg/kg	0.24–0.83	64	ATR	4000–650	SD	PLSR	R_cv_^2^ = 0.00 RMSECV = 0.08	[90]
Linolenic acid	mg/kg	0.6–1.0	104	ATR	4000–700	2DSG	PLSR	R_v_^2^ = 0.97 RMSEV = 0.02	[88]
Palmitoleic acid	mg/kg	0.13–1.42	64	ATR	4000–650	SD	PLSR	R_cv_^2^ = 0.52 RMSECV = 0.18	[90]
Chlorophyll *a*	mg/kg	0.01–0.26	52	ATR	4000–650	2D	PLSR	R_v_^2^ = 0.18 RMSEV = 0.02 RPD = 0.9	[83]
Chlorophyll *b*	mg/kg	0.10–1.70	52	ATR	4000–650	2D	PLSR	R_v_^2^ = 0.24 RMSEV = 0.37 RPD = 1.1	[83]
Lutein	mg/kg	0.60–6.29	52	ATR	4000–650	2D	PLSR	R_v_^2^ = 0.41 RMSEV = 1.27 RPD = 1.2	[83]
Chlorophylls	mg/kg	1.075–7.210	123	ATR	4000–700	Normalization	PLSR	R_v_^2^ = 0.93 RMSECV = 0.23 RPD = 4.10	[93]
Chlorophylls	mg/kg	0.51–8.84	64	ATR	4000–650	SD	PLSR	R_cv_^2^ = 0.69 RMSECV = 0.95	[90]
Chlorophylls	mg/kg	0.29–5.64	70	ATR	4000–700	SD	PLSR	R_v_^2^ = 0.97 RMSEV = 0.22	[94]
Chlorophylls	mg/kg	0.29–5.64	70	ATR	4000–700	SD	PCR	R_v_^2^ = 0.32 RMSEV = 1.61	[94]
Chlorophylls	mg/kg	0.29–5.64	70	ATR	4000–700	SD	SVM	R_v_^2^ = 0.51 RMSEV = 1.43	[94]
Carotenoids	mg/kg	0.11–25.63	64	ATR	4000–700	SD	PLSR	R_cv_^2^ = 0.46 RMSECV = 3.01	[90]
Squalene	g/kg	3.25–12.54	50	ATR	4000–600	1D + 2D	PLSR	RMSEC = 0.271 RMSEV = 0.457	[95]

Notes: R_c_^2^: multiple coefficient of determination of calibration; R_v_^2^: multiple coefficient of determination of validation; R_cv_^2^: multiple coefficient of determination of cross validation; RMSEC: root mean square error of calibration; RMSEV: root mean square error of validation test (internal); RMSECV: root mean square error of cross validation; RPD: residual prediction deviation; PLSR: partial least squares regression; PCR: principal component regression; SVM: support vector machine; TT: total tocopherols; TPC: total phenolic compounds; TPPC: total polar phenolic compounds; FD: first derivative; SD: second derivative; SNV: standard normal variate; SG: Savitzky–Golay; 2DSG: second derivative Savitzsky–Golay; WA: wavelet analysis; OSC: orthogonal signal correction.

Compared to phenolic compounds, the prediction of unsaturated fatty acids using MIRS generally yields more favorable results. As shown in Table 4, several studies have reported R^2^ values exceeding 0.9 when using MIRS combined with PLSR technology for the quantitative detection of oleic acid and linoleic acid [90,91,92], indicating high accuracy and effectiveness of this method for detecting these unsaturated fatty acids. This success is primarily due to the relatively simple molecular structure of oleic acid and linoleic acid as long-chain hydrocarbons [96], which results in consistent spectral features across samples. Such consistency facilitates the ability of model to capture molecular structure variations and establish strong regression relationships with their concentrations. In contrast, phenolic compounds exhibit more complex structures, with varying substituents and aromatic ring configurations, leading to greater variability in spectral features across different samples and increased modeling complexity [97]. As for linolenic acid, its prediction performance has shown significant variation across different studies. Research [92] employing a PLSR model for linolenic acid reported a moderate predictive ability, with an R_cv_^2^ value of 0.64. This study employed orthogonal signal correction (OSC) and wavelet analysis (WA) as spectral preprocessing methods, which aimed to remove noise and irrelevant variations from the spectral data to enhance model performance. Despite these efforts, the predictive ability of the model remained limited, possibly due to the weak spectral signals of linolenic acid or significant overlap with signals from other components, resulting in the inability of the model to fully capture the characteristic optical signals of linolenic acid even after preprocessing. Another investigation [90] applied second derivative (SD) spectral preprocessing, yet the PLSR model produced an R^2^ value of 0.00, indicating a complete failure in effectively predicting linolenic acid concentrations. This suggests that under the experimental conditions of this study, SD preprocessing was insufficient to enhance the spectral signals of linolenic acid, leaving the model unable to extract any meaningful features related to its concentration. Other potential factors contributing to the failure of the model include the diversity of samples or limitations in experimental conditions such as spectral range and resolution. By contrast, a study achieved a significantly improved prediction performance, with an R^2^ value of 0.97, indicating exceptionally high predictive accuracy [88]. The key to this success was the use of second derivative processing combined with advanced spectral preprocessing techniques (2DSG), which effectively enhanced the identification of features related to linolenic acid concentration. Additionally, compared to other studies using MIRS to determine bio-active compounds in VOO, the use of a larger number of samples (n = 104) was another crucial factor contributing to the superior performance of the prediction model.

When MIRS is applied to other bio-active compounds with significant antioxidant properties, such as carotenoids, chlorophyll, and squalene, the development of chemometric models presents varying levels of challenges. In one study, the PLSR prediction models developed for chlorophyll *a* and *b* exhibited extremely poor precision, with R_v_^2^ values of 0.18 and 0.24 [83], respectively. Despite using SD preprocessing in the study, the signal discernibility was not significantly improved, likely due to the low concentration of chlorophyll in the samples and the overlapping signals from other components. In the same study, the prediction model for lutein performed somewhat better, with an R_v_^2^ value of 0.41. However, the high RMSEV value (1.27 mg/kg) indicated that the prediction accuracy still needed improvement, and the low RPD value (1.2) suggested that the model lacked robustness and had poor generalization capability, failing to meet the requirements for practical application. In contrast, a PLSR model for total chlorophyll in another study demonstrated significantly higher predictive accuracy, with an R_v_^2^ value of 0.93 [93]. In further research conducted in 2024, optimizing the preprocessing method (using the SD algorithm) increased the R_v_^2^ value of the model to 0.97, underscoring the importance of selecting appropriate spectral preprocessing techniques to enhance model performance. Meanwhile, in the same study, the PCR and support vector machine (SVM) models for predicting chlorophyll yielded poor results, with R_v_^2^ values of 0.32 and 0.51, respectively, highlighting the critical role of applying suitable modeling algorithms when dealing with complex samples. For squalene, although the R² value was not reported in a related study [95], the combination of 1D and 2D preprocessing significantly reduced RMSECV and RMSEV values, demonstrating the importance of enhancing spectral signal clarity to improve the predictive power of the model.

Overall, these findings indicate that the effectiveness of quantitative prediction using MIRS combined with chemometrics for different bio-active compounds varies significantly depending on spectral characteristics, sample preparation methods, and preprocessing techniques. While the quantification of unsaturated fatty acids and squalene has shown relatively good results, the prediction of most individual phenolic compounds, as well as chlorophylls and carotenoids, continues to face significant challenges. This underscores the need for further optimization in experimental design and data processing in future research to enhance the accuracy of models in quantifying compounds present at low concentrations and those with complex spectral signals.

## 5. Raman Spectroscopy

Raman spectroscopy is a spectroscopic technique based on the inelastic scattering of light caused by molecular vibrations, rotations, and other low-frequency modes [98]. It is often paired with infrared spectroscopy (IR) as two principal methods for the vibrational analysis of chemical structures. Although both Raman and IR spectroscopy provide molecular vibrational information to characterize the chemical structure of substances, their detection mechanisms differ. Infrared spectroscopy relies on the absorption of specific wavelengths of infrared light by molecules, while Raman spectroscopy is based on the inelastic scattering of photons as they interact with molecules. This distinction allows Raman spectroscopy to function effectively without interference from water [99], making it particularly advantageous for the analysis of aqueous systems and biological samples, where it offers unique benefits.

Raman spectroscopy provides complementary molecular structural information compared to infrared (IR) spectroscopy due to the differences in their molecular response mechanisms. Raman spectroscopy is particularly well-suited for studying molecules with high symmetry and those that exhibit weak or no significant IR absorption [100], while IR spectroscopy is more sensitive to polar molecules. During Raman spectroscopy measurements, photons interact with the sample molecules, and the majority of photons are scattered with the same energy as the incident light. This phenomenon is known as Rayleigh scattering, which does not involve transitions in the molecular vibrational or rotational energy levels and, therefore, does not provide information about molecular vibrations [101].

**Figure 5:**
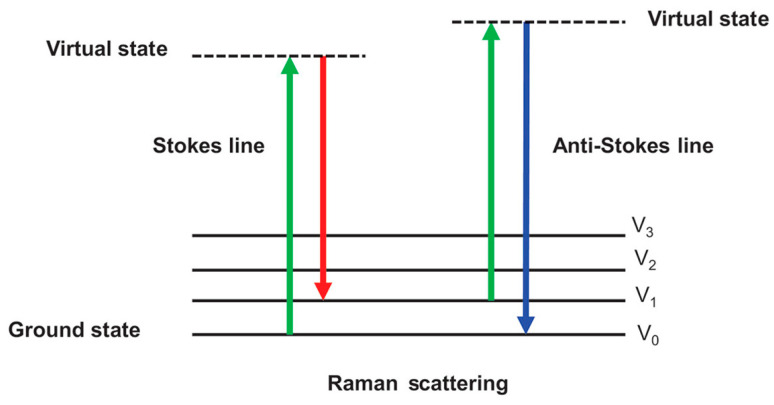
Energy level diagram for Raman scattering. (Figure from [102]).

In contrast, Raman scattering is an inelastic scattering process in which a small fraction of photons experiences a change in energy after interacting with the molecules. This energy change (Figure 5) directly reflects the characteristic vibrational modes of the molecules and provides detailed molecular structural information. Due to this energy shift, Raman spectroscopy can detect vibrational modes that are inaccessible to IR spectroscopy, making it a valuable tool in molecular identification and quantitative analysis.

Despite its advantages, particularly in the detection of non-polar molecules and complex organic compounds, Raman spectroscopy has several limitations compared to infrared (IR) spectroscopy. One major drawback is that Raman scattering is an inherently weak phenomenon, with only a small fraction of photons contributing to the scattering process [103], resulting in typically low signal intensity. Additionally, Raman spectroscopy is highly sensitive to fluorescence interference [104], certain samples may produce a strong fluorescence background that can significantly overshadow the Raman signal. In contrast, IR spectroscopy is less prone to such interference, making it suitable for a wider range of samples. Furthermore, the equipment required for Raman spectroscopy is more complex and expensive, especially the high-precision lasers, filters, and detectors, which often leads to higher costs compared to IR spectrometers.

In practical applications, a Raman spectrometer typically consists of several key components. A laser serves as the light source, with s solid-state lasers of various wavelengths, such as 532 nm or 785 nm, being used. The laser beam is focused onto the sample through a microscope, and the scattered light is collected by a lens and then spectrally separated using a monochromator or interferometer. However, since the Raman scattering signal is often overshadowed by the stronger Rayleigh scattering, precise optical filters are required to distinguish and detect the Raman signal. The filtered signal is then detected by a detector, such as a charge-coupled device (CCD) or a photomultiplier tube (PMT) [105].

To improve sensitivity and the signal-to-noise ratio, especially when detecting low-concentration or weakly Raman-active samples, Raman signal enhancement techniques are often employed. One common method is surface-enhanced Raman spectroscopy (SERS), which significantly amplifies the Raman signal by combining the sample with metallic nanoparticles (such as gold or silver) and utilizing surface plasmon resonance effects [106]. Another approach is resonant Raman spectroscopy (RRS), which enhances the signal of specific vibrational modes by selecting a laser wavelength that matches the absorption peak of the molecule [107]. Additionally, nonlinear optical techniques, such as coherent anti-Stokes Raman scattering (CARS) and stimulated Raman scattering (SRS), have shown great potential for further improving detection sensitivity and specificity.

In the Raman spectrum of VOO, various bio-active compounds generate specific Raman shifts within certain wavenumber ranges, corresponding to so-called characteristic peaks. A Raman shift represents the energy difference between the incident and scattered light, typically expressed in wavenumbers (cm^−1^). This shift reflects particular vibrational modes within the molecule. For instance, phenolic compounds exhibit a Raman shift around 1237 cm^−1^ in the Raman spectrum, corresponding to the stretching vibration caused by C=O. Some unsaturated fatty acids, such as oleic and linoleic acids, display strong Raman signals near 1270 cm^−1^ and 1306 cm^−1^, which correspond to the stretching vibrations of C–H and C–O bonds, respectively. Additionally, carotenoids, which are important antioxidants in VOO and also influence the oil color, typically show a Raman shift around 1523 cm^−1^, reflecting the vibrational modes of their conjugated C=C double bonds. Specific Raman shifts of other bio-active compounds in olive oil can be found in Table 5.

The primary bio-active components detectable in VOO using Raman spectroscopy include phenolic compounds, carotenoids, fatty acids, and so on. By measuring the area of characteristic peaks in the Raman spectra of these components, their relative concentrations can be calculated. For instance, research conducted by fitting Raman spectra with Lorentzian functions has successfully quantified carotenoids, oleic acid, and phenolic compounds in VOO samples from different regions [110]. Additionally, the relationship between the relative intensities of vibrational bands at 1660 cm^−1^ and 1445 cm^−1^ and the concentrations of oleic and linoleic acids was explored, resulting in an empirical model for assessing the relative content of these compounds [112]. Despite the potential of the predictive model, the small sample size (n = 5) and its theoretical limitations in capturing the full complexity of lipid composition may restrict its broader application. Furthermore, chemometric techniques, particularly PLSR, have been applied to quantify bio-active components based on Raman spectra. A regression model (RMSEP = 0.29%) for predicting free fatty acid content was developed using FT-Raman spectroscopy and PLSR, demonstrating its utility in production quality control, even though the accuracy was slightly lower than traditional reference methods [113].

In addition to conventional Raman methods, Raman enhancement techniques such as RRS and SERS have significantly improved sensitivity in detecting trace amounts of bio-active components in VOO. For instance, RRS has been shown to effectively enhance the weak signals of carotenoids, enabling rapid and accurate quantification [111]. This technique has been particularly useful in monitoring carotenoid degradation during storage and heating, which directly affects the quality of oil. Likewise, RRS has been employed to analyze standard solutions of lutein and β-carotene at various concentrations, establishing a correlation between compound concentration and the intensity and position of characteristic vibrational modes in the Raman spectra [109]. This approach provided a fast and straightforward method for quantitatively analyzing the ratio of lutein to β-carotene in VOO. Additionally, SERS has been applied to monitor bio-active components in VOO, particularly useful for analyzing materials with weak Raman signals or strong fluorescence backgrounds [110]. By processing the spectral data with a wavelet transform algorithm, fluorescence interference was effectively eliminated, improving the clarity and readability of the Raman signals. However, the need for specific metallic nanostructures as an enhancement substrate in SERS introduces additional complexity and cost to the sample preparation process.

Future research should prioritize refining SERS and other enhancement techniques to further increase sensitivity and reduce interference. This would involve developing more consistent nanoparticle substrates, exploring new materials, and improving the reproducibility of SERS signals. Additionally, incorporating machine learning algorithms into the analysis of Raman spectra could enable a more precise quantification of bio-active components by automating the interpretation of complex spectral data. These advancements would not only enhance the detection of key antioxidants in VOO but also broaden the use of Raman spectroscopy for other functional foods.

## 6. Conclusions and Outlook

In summary, vibrational spectroscopic techniques, including near-infrared (NIR), mid-infrared (MIR), and Raman spectroscopies, have demonstrated considerable potential in the detection and quantification of bio-active compounds in VOO. Each technique offers unique advantages and limitations. NIRS is suitable for rapid quantitative analysis, particularly in large-scale industrial applications, but it is less specific in identifying individual compounds compared to MIR and Raman spectroscopy. MIR spectroscopy excels in providing detailed molecular information due to its ability to detect fundamental vibrational modes, making it highly effective for analyzing complex mixtures of compounds. Raman spectroscopy, especially when enhanced through techniques like SERS and RRS, has proven valuable for detecting compounds in low concentrations and materials that are challenging to analyze due to fluorescence or weak signal responses.

Even though NIRS and MIRS are widely applied in the detection of bio-active compounds in VOO, their future potential remains significant, particularly as they become more deeply integrated with the rapidly advancing fields of deep learning, data fusion, and advanced chemometric techniques. By leveraging the power of deep learning algorithms, these methods could significantly enhance predictive accuracy, enabling more precise and efficient analysis of complex bio-active compositions. Additionally, the integration of data fusion, which involves combining multiple sources of spectral data, could further enhance the robustness and reliability of detection, addressing the limitations of individual techniques. Moreover, the future of Raman spectroscopy lies in the development of surface-enhanced techniques, especially through innovations in nanomaterials. Advancing materials for SERS could greatly improve sensitivity and enable the detection of trace bio-active compounds. 

As technology continues to evolve, it is anticipated that these spectroscopic methods will not only expand their role in quality control and the authentication of VOO but also contribute significantly to the broader field of functional food analysis, thereby supporting the growing consumer demand for health-promoting natural products.

## Figures and Tables

**Table 1:** Functional group assignment for selected NIR wavelengths (table adapted from [58]).

Selected Wavelength (nm)	Wavenumber (cm^−1^)	Functional Groups	Assignment	Reference
1167	8569	–CH_3_	C–H stretch second overtone	[58]
1208	8278	–CH_2_	C–H stretch second overtone	[50]
1220	8197	HC=CH–	C–H stretch second overtone	[59]
1373	7283	–CH_3_	2C–H stretch + C–H deformation	[58]
1400	7143	–OH	O–H stretch	[55]
1462	6840	–CH_2_	2C–H stretch + C–H deformation	[58]
1720	5814	–CH_2_, –CH_3_, =CH_2_	C–H first overtone	[52]
1760	5682	–CH_2_, –CH_3_, =CH_2_	C–H first overtone	[51]
1832	5459	–COOR	C–H first overtone	[54]
1844	5423	–CH_2_	C–H first overtone	[58]
1950	5128	–OH	O–H stretch first overtone	[55]
2022	4946	–COOR	C–H str. + C=O str.	[58]
2049	4880	–COOR	C–H str. + C=O str.	[58]
2144	4664	HC=CH–	C–H str. + C=C str.	[54]

**Table 2:** Developed chemometrics models for various bio-active compounds in VOO using different unit ranges, NIR spectral intervals, spectral pre-processing methods, and optical path lengths.

Analytes	Units	Range	Samples Number	Wavelength Range (nm)	Path Length (mm)	Spectra Preprocessing	Statistical Methods	Results	Reference
TPC	mg/kg	110.73–593.95	97	800–2500	8	FD + SD + MSC	PLSR	R_v_^2^ = 0.79 RMSEV = 44.50 RPD = 1.71	[57]
TPC	mg/kg	44.49–738.76	98	978–2500	0.5	MSC	PLSR	R_v_^2^ = 0.34 RMSEV = 82.10 RPD = 1.24	[62]
TPC	mg/kg	44.49–738.76	98	1100–2500	0.2	MSC	PLSR	R_v_^2^ = 0.21 RMSEV = 89.66 RPD = 1.13	[62]
TPPC	mg/kg	13.4–946.7	93	800–2500	8	FD + SNV	PLSR	R_v_^2^ = 0.82 RMSEV = 76.70 RPD = 2.36	[72]
Hydroxytyrosol	mg/kg	0.3–42.9	93	800–2500	8	FD + SNV	PLSR	R_v_^2^ = 0.55 RMSEV = 4.84 RPD = 1.25	[72]
Hydroxytyrosol	mg/kg	1.07–36.12	97	800–2500	8	FD + SD + MSC	PLSR	R_v_^2^ = 0.20 RMSEV = 4.06 RPD = 1.03	[57]
Tyrosol	mg/kg	1.2–32.8	93	800–2500	8	FD + SNV	PLSR	R_v_^2^ = 0.55 RMSEV = 5.27 RPD = 1.43	[72]
Tyrosol	mg/kg	1.57–64.39	97	800–2500	8	FD + SD + MSC	PLSR	R_v_^2^ = 0.34 RMSEV = 3.20 RPD = 1.06	[57]
Hydroxytyrosol derivatives	mg/kg	21.41–380.37	97	800–2500	8	FD + SD + MSC	PLSR	R_v_^2^ = 0.85 RMSEV = 25.50 RPD = 1.99	[57]
Hydroxytyrosol derivatives	mg/kg	40.54–75.20	93	800–2500	8	FD + SNV	PLSR	R_v_^2^ = 0.82 RMSEV = 43.1 RPD = 2.39	[72]
Tyrosol derivatives	mg/kg	68.33–315.92	97	800–2500	8	FD + SD + MSC	PLSR	R_v_^2^ = 0.57 RMSEV = 23.80 RPD = 1.23	[57]
Tyrosol derivatives	mg/kg	61.1–456.1	93	800–2500	8	FD + SNV	PLSR	R_v_^2^ = 0.84 RMSEV = 41.5 RPD = 2.31	[72]
Oleuropein	mg/kg	–	97	800–2500	8	FD + SD + MSC	PLSR	R_v_^2^ = 0.88 RMSEV = 179.00 RPD = 2.13	[57]
α-tocopherol	mg/kg	90.96–249.33	97	800–2500	8	FD + SD + MSC	PLSR	R_v_^2^ = 0.60 RMSEV = 15.20 RPD = 1.30	[57]
α-tocopherol	mg/kg	54.50–755.90	206	1100–2300	10	1DSG + 2DSG	PLSR	R_cv_ = 0.91 SEC = 36.14	[55]
β-tocopherol	mg/kg	9.11–17.20	97	800–2500	8	FD + SD + MSC	PLSR	R_v_^2^ = 0.14 RMSEV = 1.53 RPD = 1.04	[57]
β-tocopherol	mg/kg	0.7–14.1	211	1100–2300	10	1DSG + 2DSG	PLSR	R_cv_ = 0.52 SEC = 0.58	[55]
γ-tocopherol	mg/kg	10.73–36.56	97	800–2500	8	FD + SD + MSC	PLSR	R_v_^2^ = 0.40 RMSEV = 2.23 RPD = 1.17	[57]
γ-tocopherol	mg/kg	2.5–103.8	211	1100–2300	10	1DSG + 2DSG	PLSR	R_cv_ = 0.88 SEC = 5.34	[55]
Total tocopherols	mg/kg	110.8–278.8	97	800–2500	8	FD + SD + MSC	PLSR	R_v_^2^ = 0.44 RMSEV = 19.30 RPD = 1.17	[57]
Total tocopherols	mg/kg	64.2–1078.0	213	1100–2300	10	1DSG + 2DSG	PLSR	R_cv_^2^ = 0.88 SEC = 57.15	[55]
Linoleic acid	%	0.00–15.68	104	978–2500	0.5	MSC	PLSR	R_v_^2^ = 0.88 RMSEV = 0.83 RPD = 2.81	[62]
Linoleic acid	%	0.00–15.68	104	1100–2500	0.2	MSC	PLSR	R_v_^2^ = 0.90 RMSEV = 0.83 RPD = 2.81	[62]
Linoleic acid	%	4.39–24.83	73	833–2500	8	SD	PLSR	R_v_^2^ = 0.99 RMSEV = 0.23 RPD = 16.00	[70]
Linoleic acid	%	3.00–22.00	82	772–2222	8	SNV	PLSR	R_v_^2^ = 0.99 RMSEV = 0.46 RPD = 8.80	[71]
Linoleic acid	%	3.31–41.90	25	900–1700	8	SNV + SG	PLSR	R_v_^2^ = 0.92 RMSEV = 0.57	[69]
Linoleic acid	%	3.31–41.90	25	1350–2150	8	SNV + SG	PLSR	R_v_^2^ = 0.72 RMSEV = 0.76	[69]
Linolenic acid	%	0.44–1.79	73	833–2500	8	FD + SNV	PLSR	R_v_^2^ = 0.85 RMSEV = 0.08 RPD = 2.80	[70]
Oleic acid	%	45.07–80.57	25	900–1700	8	SNV + SG	PLSR	R_v_^2^ = 0.86 RMSEV = 1.46	[69]
Oleic acid	%	45.07–80.57	25	1350–2150	8	SNV + SG	PLSR	R_v_^2^ = 0.58 RMSEV = 2.35	[69]
Oleic acid	%	58.90–77.90	104	978–2500	0.5	MSC	PLSR	R_v_^2^ = 0.56 RMSEV = 1.47 RPD = 1.50	[62]
Oleic acid	%	58.90–77.90	104	1100–2500	0.2	MSC	PLSR	R_v_^2^ = 0.53 RMSEV = 1.53 RPD = 1.44	[62]
Oleic acid	%	49.14–79.69	73	833–2500	8	FD + SLS	PLSR	R_v_^2^ = 0.99 RMSEV = 0.28 RPD = 17.60	[70]
Oleic acid	%	56.00–80.00	82	772–2222	8	SNV	PLSR	R_v_^2^ = 0.96 RMSEV = 1.03 RPD = 4.70	[71]
Cholesterol	%	0.00–1.35	73	833–2500	8	COE	PLSR	R_v_^2^ = 0.42 RMSEV = 0.14 RPD = 1.32	[70]
Campesterol	%	13.2–3.99	73	833–2500	8	MSC	PLSR	R_v_^2^ = 0.17 RMSEV = 0.57 RPD = 1.14	[70]
Stigmasterol	%	0.17–1.88	73	833–2500	8	SLS	PLSR	R_v_^2^ = 0.22 RMSEV = 0.35 RPD = 1.14	[70]
β-sitosterol	%	45.94–89.66	73	833–2500	8	FD + MSC	PLSR	R_v_^2^ = 0.40 RMSEV = 6.41 RPD = 1.30	[70]
Δ5-avenasterol	%	3.28–17.98	73	833–2500	8	FD	PLSR	R_v_^2^ = 0.27 RMSEV = 2.77 RPD = 1.17	[70]
Total sterol	mg/kg	687.9–3087.4	73	833–2500	8	FD + MSC	PLSR	R_v_^2^ = 0.84 RMSEV = 192.00 RPD = 2.64	[70]
Chlorophylls	mg/kg	0.082–25.23	97	978–2500	0.5	MSC	PLSR	R_v_^2^ = 0.31 RMSEV = 4.42 RPD = 1.20	[62]
Chlorophylls	mg/kg	0.082–25.23	97	1100–2500	0.2	MSC	PLSR	R_v_^2^ = 0.56 RMSEV = 3.58 RPD = 1.49	[62]
Chlorophylls	mg/kg	0.70–27.50	183	450–2500	1	DT	PLSR	R_c_^2^ = 0.99 RMSEV = 0.66 RPD = 7.70	[68]
Chlorophylls	mg/kg	1.40–88.10	255	1100–2500	5	SG	PLSR	R_c_^2^ = 0.56	[67]
Chlorophylls	mg/kg	1.40–88.10	255	350–2500	5	SG	PLSR	R_c_^2^ = 0.96 RMSEV = 3.50 RPD = 4.10	[67]
Carotenoids	mg/kg	0.12–13.13	96	978–2500	0.5	MSC	PLSR	R_v_^2^ = 0.52 RMSEV = 1.35 RPD = 1.44	[62]
Carotenoids	mg/kg	0.12–13.13	96	1100–2500	0.2	MSC	PLSR	R_v_^2^ = 0.66 RMSEV = 1.14 RPD = 1.71	[62]
Carotenoids	mg/kg	1.60–18.10	183	450–2500	1	DT	PLSR	R_c_^2^ = 0.99 RMSEV = 0.96 RPD = 5.20	[68]
Carotenoids	mg/kg	2.10–38.50	255	1100–2500	5	SG	PLSR	R_c_^2^ = 0.62	[67]
Carotenoids	mg/kg	2.10–38.50	255	350–2500	5	SG	PLSR	R_c_^2^ = 0.95 RMSEV = 1.80 RPD = 3.90	[67]
Squalene	g/kg	1.00–10.10	177	1100–2300	–	MN + SNV + 1DSG + 2DSG	PLSR	R_c_^2^ = 0.86 RMSEV = 1.20 RPD = 2.30	[73]
Squalene	g/kg	1.00–10.10	177	350–2500	10	MN + SNV + 1DSG + 2DSG	PLSR	R_c_^2^ = 0.76 RMSEV = 1.00 RPD = 1.90	[73]

Notes: R_c_^2^: multiple coefficient of determination of calibration; R_v_^2^: multiple coefficient of determination of validation; R_cv_^2^: multiple coefficient of determination of cross validation; RMSEC: root mean square error of calibration; RMSEV: root mean square error of validation test (internal); RMSECV: root mean square error of cross validation; SEC: standard error of calibration; RPD: residual prediction deviation; PLSR: partial least squares regression; TT: total tocopherols; TPC: total phenolic compounds; TPPC: total polar phenolic compounds; FD: first derivative; SD: second derivative; SNV: standard normal variate; MSC: multiplicative scatter correction; SLS: straight line subtraction; COE: constant offset elimination; MN: mean normalization; SG: Savitzky–Golay; DT: derivative transformation; 1DSG: first derivative Savitzsky–Golay; 2DSG: second derivative Savitzsky–Golay.

**Table 5:** Main Raman shift observed in the spectra with assignments.

Analytes	Raman Shift (cm^−1^)	Associated Chemical Bond/Structure	Reference
Hydroxytyrosol	780	O–H bending	[108]
Carotenoids	1004	C–CH_3_ bending	[109]
Carotenoids	1150, 1525	C=C stretching, C–H bending	[110]
Carotenoids	1156	C–C stretching	[111]
TPC	1237	C–O stretching	[110]
Oleic and linoleic acid	1270	In-phase C–H bending	[112]
Oleic and linoleic acid	1306	–CH_2_ torsional bending	[112]
Oleic acid	1350	C–H bending	[110]
Oleic acid	1442, 1655	C=C stretching	[113]
Carotenoids	1523	C=C stretching	[109]

Notes: TPC: total phenolic compounds.

## Data Availability

No new data were created or analyzed in this study. Data sharing is not applicable to this article.
